# Water extract of Cnidii Rhizoma suppresses RANKL-induced osteoclastogenesis in RAW 264.7 cell by inhibiting NFATc1/c-Fos signaling and prevents ovariectomized bone loss in SD-rat

**DOI:** 10.1186/s12906-019-2611-8

**Published:** 2019-08-09

**Authors:** Ka-Yeon Lee, Jae-Hyun Kim, Eun-Young Kim, Mijung Yeom, Hyuk-Sang Jung, Youngjoo Sohn

**Affiliations:** 1 Department of Anatomy, College of Korean Medicine, Kyung Hee University, Seoul, 02447 Republic of Korea; 2 Acupuncture and Meridian Science Research Center, College of Korean Medicine, Kyung Hee University, Seoul, 02447 Republic of Korea

**Keywords:** Cnidii Rhizoma, Osteoclast, NFATc1, c-Fos, Ovariectomized-rat

## Abstract

**Background:**

Cnidii Rhizoma is the dried root stem of *Cnidium officinale* Makino. Cnidii Rhizoma (CR) has been used to treat menstrual irregularity, menstrual pain, and menopause in Korea. However, the effects and mechanisms of CR on RANKL-induced osteoclastogenesis pathway remain to be elucidated. In this study, we investigated the effects of CR on the inhibition of bone resorption of osteoclast and its mechanism RANK signaling pathway.

**Methods:**

The anti-osteoclastogenesis of water extract of CR was measured using RAW 264.7 cell. Tartrate-resistant acid phosphatase (TRAP) assay, pit assay, reverse transcription polymerase chain reaction (RT-PCR) and western blot were performed. Moreover, the effects of CR were determined with an in vivo model using ovariectomized (OVX) rats.

**Results:**

CR extract suppressed osteoclastogenesis, its activity and bone resorption activity through decreasing gene of osteoclast-related such as nuclear factor of activated T-cells, cytoplasmic 1 (NFATc1), c-Fos, etc. Moreover, CR extract prevented the bone loss in OVX rats.

**Conclusion:**

These results show that CR has a positive effect on menopausal osteoporosis by suppressing osteoclastogenesis.

**Electronic supplementary material:**

The online version of this article (10.1186/s12906-019-2611-8) contains supplementary material, which is available to authorized users.

## Background

Osteoporosis is a critical diseases characterized by bone loss and impaired bone quality that can lead to an increased risk of fracture [1, 2]. In normal skeleton, bone remodeling occurs through bone resorption by osteoclasts and the synthesis of bone by osteoblasts. An imbalance in these processes can cause osteoporosis [3–5]. Osteoclasts are multinucleated giant cells that can resorb bone matrix. Osteoclasts can differentiate for many reasons, one of which is a deficiency of a sexual hormone such as estrogen, which leads to menopause, increased bone resorption activity by osteoclasts, and the increased formation of osteoclasts, and these factors have key roles in bone loss [6, 7]. Therefore, osteoporosis can be treated by suppressing the activation of osteoclasts.

Osteoclasts are generated by the fusion of a monocyte and macrophage derived from osteoclast precursors induced by osteoclast cytokines, such as the receptor activator of the nuclear factor kappa B (NF-κB) ligand (RANKL). There are many experimental methods for osteoclastogenesis, such as using RAW 264.7 cells and BMM cells derived from mice [8, 9]. Among these methods, the use of the RAW 264.7 murine cell line has been demonstrated to be a significant tool for in vitro researches of osteoclastogenesis and its activity [10]. RANKL can conjugate to its receptor RANK which is located on the RAW 264.7 cell surface [11]. This conjugation leads to the expression of tumor necrosis factor receptor-associated factor 6 (TRAF6). This results in the activation of downstream signaling cascades including the nuclear factor of activated T-cells, cytoplasmic 1 (NFATc1) and c-Fos. Both transcription factors have critical and specific roles in osteoclastogenesis [12]. Moreover, NFATc1 can directly control osteoclast specific genes [5, 13–15].

Cnidii Rhizoma (CR) is the dried root stem of *Cnidii officinale* Makino, which is called “Chunkung” in Korea [16]. In oriental medicine, CR has been used to treat menstrual irregularity, menstrual pain, and menopause for woman [17, 18]. In previous study, CR has been reported to have various biological activities such as angiogenesis [19], anti-cancer [20, 21], anti-oxidant [22] and anti-inflammatory effect [23]. Also, various studies have shown that anti-inflammatory and anti-oxidant effects are associated with osteoclast inhibition [24–26]. Recently, Ligusticum chuanxiong which is observed that the same chemical type of compound constitutes the main component of CR [27] has been shown to be effective in osteoblast activity [28]. Inhibition of osteoclast differentiation is more important than promoting osteoblast differentiation on postmenopausal osteoporosis [5]. However, studies on the effect of CR on osteoclasts have not yet been investigated. In typical anti-resorptive medicines for postmenopausal osteoporosis (bisphosphonate and hormonal therapy etc.), there are several side effects such as breast and uterine cancer, vascular disease [4, 29]. In consideration of recent studies, we expected that if CR, which is used for gynecological diseases, has effect of the inhibition for osteoclast differentiation and bone absorption in ovariectomized (OVX) osteoporosis model, it could be useful in the treatment of menopausal symptoms including postmenopausal osteoporosis.

In this study, we focused on determining whether water extract of CR suppressed osteoclastogenesis and the bone resorption activity by inhibiting RANKL induced NFATc1, c-Fos, and the RANK signaling pathway in RAW 264.7 cells. In addition, we carried out the alleviation effect in OVX rat’s bone loss.

## Methods

### Preparation of CR extracts

CR was authenticated by Professor Yungmin Bu at the Herbology Laboratory, College of Korean Medicine, Kyung Hee University and purchased from Kyung Hee University Medical Center. The extract was prepared by decocting 300 g of the dried herb with 3 L of boiling distilled water for 2 h and then filtering it using filter paper. The extract was collected in a rotary evaporator and lyophilized, which yielded 81 g of dried powder (yield ratio 27%), and stored at − 20 °C until use. A voucher specimen of the plant material used in this study has been deposited in the Department of anatomy herbarium [KHU-ANA-A061].

### Analysis of CR extract with HPLC

Standard stock solutions (1000 μg/ml) of chlorogenic acid (primary pharmaceutical reference standard, Sigma-Aldrich, Saint-Louis, MI, USA) were prepared in methanol. A Waters 2695 system equipped with a Waters 2487 Dual λ absorbance detector was used for the analysis of both chlorogenic acid from CR and chlorogenic acid as the standard. The separation was carried out on an Xbridge-C18 (250 mm × 4.6 mm, 5 μm) with a C18 guard column. The binary mobile phase consisted of solvent A, methanol, and solvent B, water containing 1% acetic acid. All the solvents were filtered through a 0.45 μm filter prior to use. The elution conditions were 0–30 min. of 15% A and 85% B at a flow rate of 1.0 ml/min with an injection volume of 10 μl. Chlorogenic acid was detected at 350 nm.

### Cell culture of the osteoclast precursor cells and cell viability

RAW 264.7 cells (Korea cell line bank, Seoul, Korea), a cell line derived from murine macrophage cells, were maintained in Dulbecco’s medium Modified Eagle (DMEM) supplemented with 10% fetal bovine serum (FBS) and antibiotics (1% penicillin/streptomycin) at 37 °C in an atmosphere containing 5% CO_2_ that 95% humidity. Cell viability of RAW 264.7 cells for CR was determined using MTS solution (Promega, Madison, WI).

### TRAP assay and bone resorption assay

For differentiation, RAW 264.7 cells were treated with RANKL (100 ng/ml) and CR for 5 days. For the TRAP staining, mature osteoclasts were washed with DPBS (Gibco, Gaithersburg, MD, USA), the differentiated RAW 264.7 cells were stained using the TRAP staining kit (Sigma Aldrich, Saint Louis, MI, USA) according to the manufacturer’s protocol. TRAP-staining positive differentiated RAW 264.7 cells were counted under a microscope (Olympus, Tokyo, Japan). Measuring of the TRAP activity was performed as previously described [9]. RAW 264.7 cells were cultured in the osteo assay strip well plate (Corning Incorporated, New York, NY, USA) with RANKL and CR. The pit of the plate was captured under an inverted microscope.

### Western blot analysis

The cells were lysed in lysis buffer (50 mM Tris. Cl, 150 mM NaCl, 1% NP-40, 0.5% Na.deoxycholate, 0.1% SDS, and a protease inhibitor cocktail, phosphatase inhibitor cocktail). The equal amounts of proteins were separated by SDS-PAGE and transferred membrane (Whatman Protran, Dassel, Germany). The membrane was blocked and then incubated with primary antibodies (1:1000) such as NFATc1 (BD Pharmingen San Diego, CA, USA), c-Fos and Actin (Santa Cruz Biotechnology, Santa Cruz, CA, USA) followed by secondary antibodies (1:10000) (Jackson ImmunoResearch, West Grove, PA, USA). The proteins attached to the membrane was measured using enhanced chemiluminescence (ECL) detection system (Santa Cruz Biotechnology, Santa Cruz, CA, USA), according to the manufacturer’s instructions.

### Gene expression analysis

Total RNA was isolated using Trizol (TaKaRa Bio, Otsu, Japan) according to the manufacturer’s protocols. The concentration of extracted RNA was measured with Nanodrop 2.0 (Thermo scientific, PA, USA) and converted into cDNA with a reverse transcription kit (Invitrogen, Carlsbad, CA, USA). The RT-PCR reaction was composed of 22–40 cycles of denaturation, annealing and extension using Taq polymerase (Kapa Biosystems, Woburn, MA, USA). The primer sequences are as follows Table 1. The reacted products were run on SYBR green stained agarose gel (Invitrogen, Carlsbad, CA, USA). The gel was photographed using gel documentation system (NαBI, Neo science, Seoul, Korea) and determined using ImageJ software (Image J1, National Institutes of Health, Bethesda, MD, USA).

**Table 1:** Primer sequence for RT-PCR analysis

Target genes	Primer sequence	Accession number	Annealing	Cycle	Base pair
TRAP	F: 5′-act tcc cca gcc ctt act acc g-3′ R: 5′-tca gca cat agc cca cac cg-3′	NM_007388.3	58 °C	30	381
NFATc1	F: 5′-tgc tcc tcc tcc tgc tgc tc-3′ R: 5′-cgt ctt cca cct cca cgt cg-3′	NM_198429.2	58 °C	32	480
c-Fos	F: 5′-atg ggc tct cct gtc aac ac-3′ R: 5′-ggc tgc caa aat aaa ctc ca-3′	NM_010234.3	58 °C	33	480
RANK	F: 5′-aaa cct tgg acc aac tgc ac-3′ R: 5′-acc atc ttc tcc tcc cga gt-3′	NM_009399.3	53 °C	32	377
CA2	F: 5′-ctc tca gga caa tgc agt gct ga-3′ R: 5′-atc cag gtc aca cat tcc agc a-3′	NM_001357334.1	58 °C	32	411
CTK	F: 5′-agg cgg cta tat gac cac tg-3′ R: 5′-ccg agc caa gag agc ata tc-3′	NM_007802.4	58 °C	27	403
MMP-9	F: 5′-cga ctt ttg tgg tct tcc cc-3′ R: 5′-tga agg ttt gga atc gac cc-3′	NM_013599.4	58 °C	33	258
CTR	F: 5′-tgc att ccc ggg ata cac ag-3′ R: 5′-agg aac gca gac ttc act gg-3′	NM_001355192.1	59 °C	40	393
GAPDH	F: 5′-act ttg tca agc tca ttt cc-3′ R: 5′-tgc agc gaa ctt tat tga tg-3′	NM_008084.3	58 °C	30	267

### In vivo model of osteoporosis and serum analysis

Forty female Sprague-Dawley (SD) rats (240-250 g) were provided by Nara Biotech (Seoul, Korea). All experiments were conducted according to the principles of the Institutional Animal Care and The protocol was approved by Committee of the Kyung Hee University Laboratory Animal Center (permission number: KHUASP (SE)-13–051). The rats were acclimatized in the laboratory environment for one week and then they were either sham-operated (*n* = 8) or ovariectomized (*n* = 32). To induce postmenopausal osteoporosis model. The ovariectomized (OVX) group removed both ovaries. In addition, the sham-operated group did not remove the ovaries after laparotomy to give the same stress. Rats were randomly placed into 5 groups (n = 8); (1) Sham, sham-operated rats and distilled water-orally administered; (2) OVX, OVX control rats and distilled water-orally administered; (3) E_2,_ OVX and 17β-estradiol (100 μg/kg)-orally administered; (4) CR-L, OVX and CR 36 mg/kg-orally administered; (5) CR-H, OVX and CR 360 mg/kg-orally administered. Oral administration was carried out every morning for 8 weeks. At the end of the treatment, rats were injected intraperitoneally with a high concentration of pentobarbital sodium (80 mg/kg) for anesthesia and then blood close to the lethal dose was collected with a cardiac puncture and cervical dislocation was progressed. The uterus and femurs and tibias were collected and weighed. The serum samples were prepared by centrifugation of the collected blood samples (2000 rpm for 10 min at 4 °C) and then stored at − 80 °C. Osteocalcin was measured by Mouse Osteocalcin ELISA Kit (LSBio, WA, USA). Measuring of the TRAP activity was performed as previously described [9].

### Histological examination

The left femur was fixed in 10% neutral buffered formalin (NBF) for 2 days, demineralized using 10% Ethylenediamine tetraacetic acid (EDTA-2Na) for 3 weeks, and then dehydrated with ethanol, clarified with xylene, and embedded with paraffin. Paraffin embedded tissue was sectioned on a rotary microtome (ZEISS, Oberkochen, GERMANY). The sectioned tissues were stained with hematoxylin–eosin (H&E). Moreover, to confirm osteoclastogenesis inhibition, TRAP staining proceeded. TRAP-stained tissues were counterstained with methyl green. The histologic changes of the femur caused by the ovariectomy were observed with a light microscope (DP73, Olympus, Tokyo, Japan) (40, 100×).

### Immunohistochemical (IHC) staining

The left femur was fixed in 10% NBF for 2 days, demineralized using 10% EDTA-2Na for 3 weeks, and then dehydrated with ethanol, clarified with xylene, and embedded with paraffin. Paraffin embedded tissue was sectioned on a rotary microtome. Endogenous peroxidase was blocked in 3% H_2_O_2_/Mt-OH for 15 min at room temperature. Then, 20 μg/ml proteinase K (Thermo fisher, PA, USA) was used for epitope retrieval for 10 min at 37 °C, for blocking, normal serum (Gibco, Gaithersburg, MD, USA) was reacted at room temperature for 30 min. The tissues were incubated with primary antibody diluted in 0.5% BSA, including anti-NFATc1 (1:100, Santa Cruz Biotechnology, CA, USA), anti-c-fos (1:100, Santa Cruz Biotechnology, CA, USA) and anti-cathepsin K (1:100, Santa Cruz Biotechnology, CA, USA), at 4 °C overnight. The tissues were then stored in 1:100 biotinylated secondary antibody (Vector Labs, Burlingame, CA, USA) for 60 min at room temperature. The tissues were incubated in horseradish peroxidase-streptavidin using ABC kit (Vector Labs, Burlingame, CA, USA) for 30 min at room temperature and stained with 3,3′-Diaminobenzidine solution (Vector Labs). The tissues were counterstained with hematoxylin, dehydrated and mounted. The immunohistochemical stained tissues were observed with a light microscope (100, 200×).

### Statistical analysis

All data are presented as mean ± S. E from at least three or more experiments. Data were evaluated by One-way analysis of variance (ANOVA) between two mean values, and this was followed by Dunnett’s multiple comparison test. *P* < 0.05 was considered statistically significant.

## Results

### Quality assessment of the CR extract

**Fig. 1:**
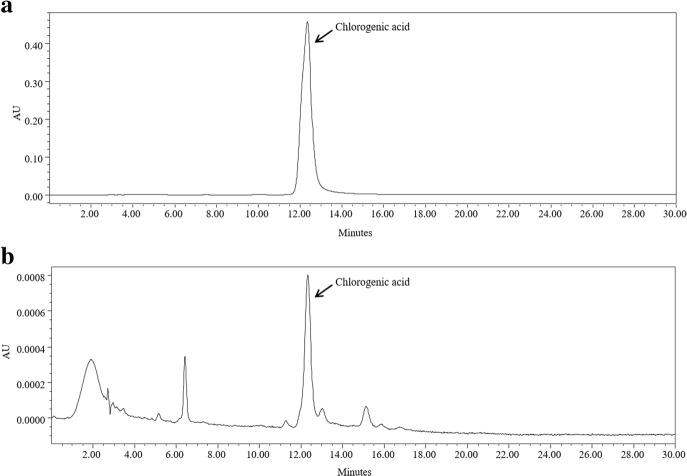
HPLC Chromatograms of the **a** Chlorogenic acid and **b** CR

Chlorogenic acid is a standard marker for the authentication of CR [30]. The chromatogram of the water extract from CR showed many peaks at a retention time of 0 and 30 min, and chlorogenic acid was found at the same retention time as the standards (Fig. 1).

### Effects of CR on cell viability

**Fig. 2:**
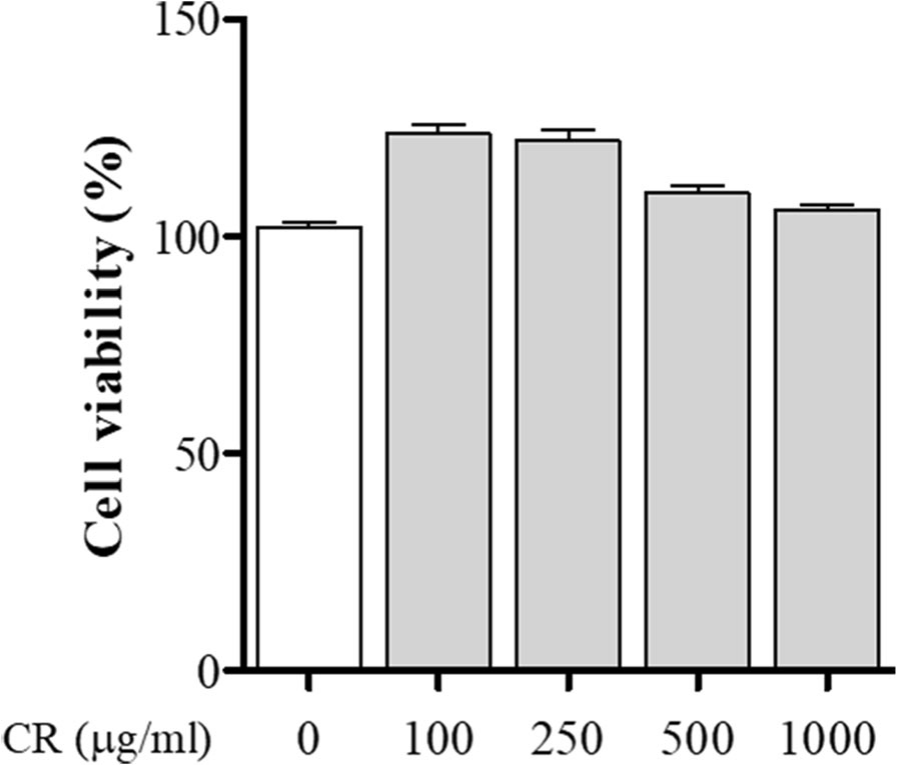
Effect of CR on RAW 264.7 cell viability was determined by MTS assay**.** Determination of cytotoxicity of CR on RAW 264.7 cells. Columns and error bars represent the mean ± S. E of three independent experiments

Before performing the in vitro tests, we determined the cell viability of CR on osteoclast precursors. All concentrations of CR were shown to have a viability equivalent to the normal. CR did not show any cytotoxicity in the RAW 264.7 cells (Fig. 2).

### CR inhibited osteoclastogenesis in RAW 264.7 cells

**Fig. 3:**
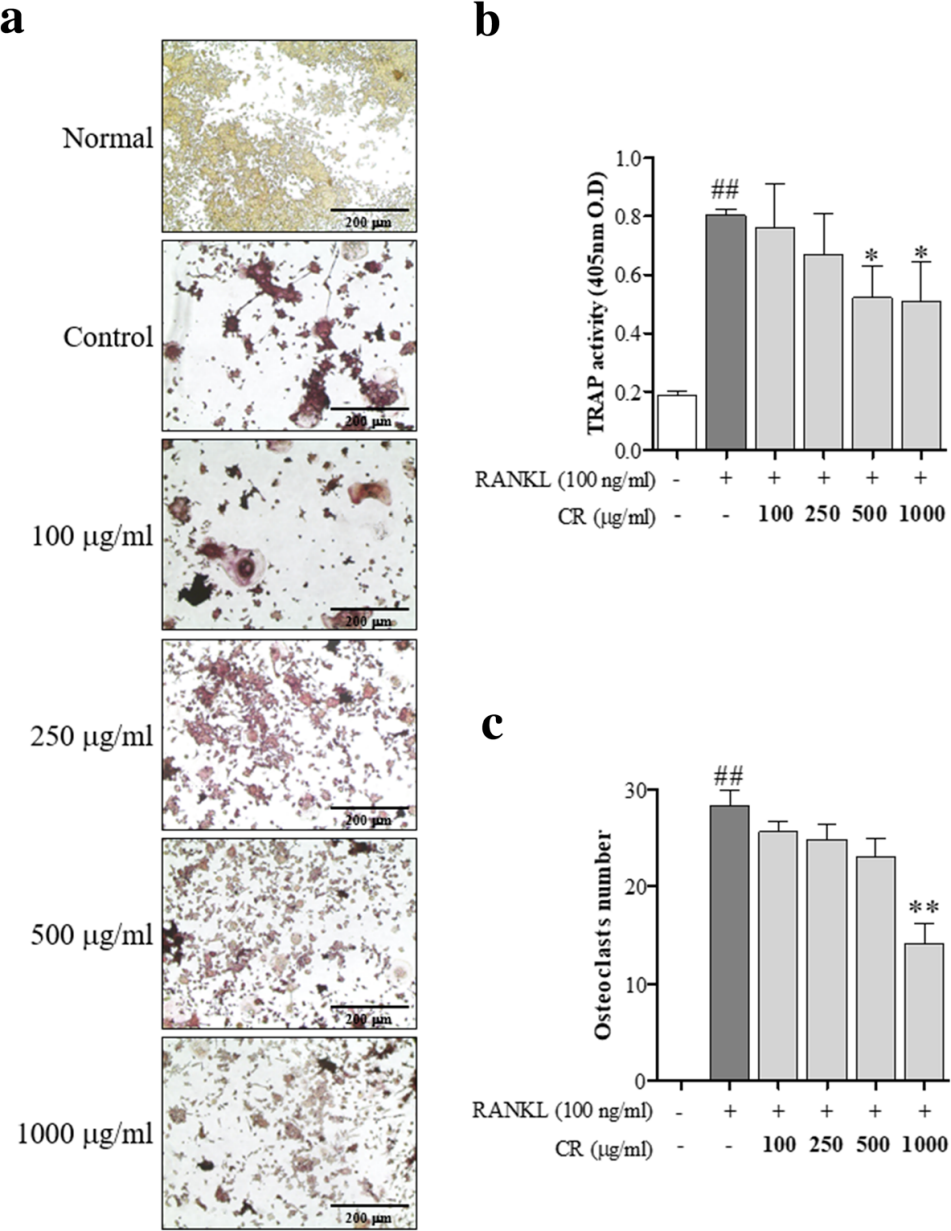
Effect of CR on osteoclast differentiation. The cells were stained with the TRAP assay kit, and media were collected for the TRAP activity. **a** TRAP-positive multinuclear cells were captured using an inverted microscope (100×, Scale bars: 200 μm). **b** Cells were counted, and **c** the media were measured for TRAP activity by an ELISA reader. Columns and error bars represent the mean ± S. E of three independent experiments. ^##^*p* < 0.01 compared with normal and ^**^p < 0.01, ^*^p < 0.05 compared with the control

To measure the effect of CR on osteoclast formation using the murine monocyte/macrophage cell line RAW 264.7, RANKL (100 ng/ml) was used to induce TRAP-positive multinucleated osteoclast differentiation in RAW 264.7 cells. CR had inhibitory effects on TRAP-positive cells in a dose-dependent manner (Fig. 3a and b). Furthermore, CR also had an inhibitory effect on the TRAP activity (Fig. 3c). These data are consistent with the inhibitory effects on osteoclast formation.

### CR inhibited the bone resorptive activity

**Fig. 4:**
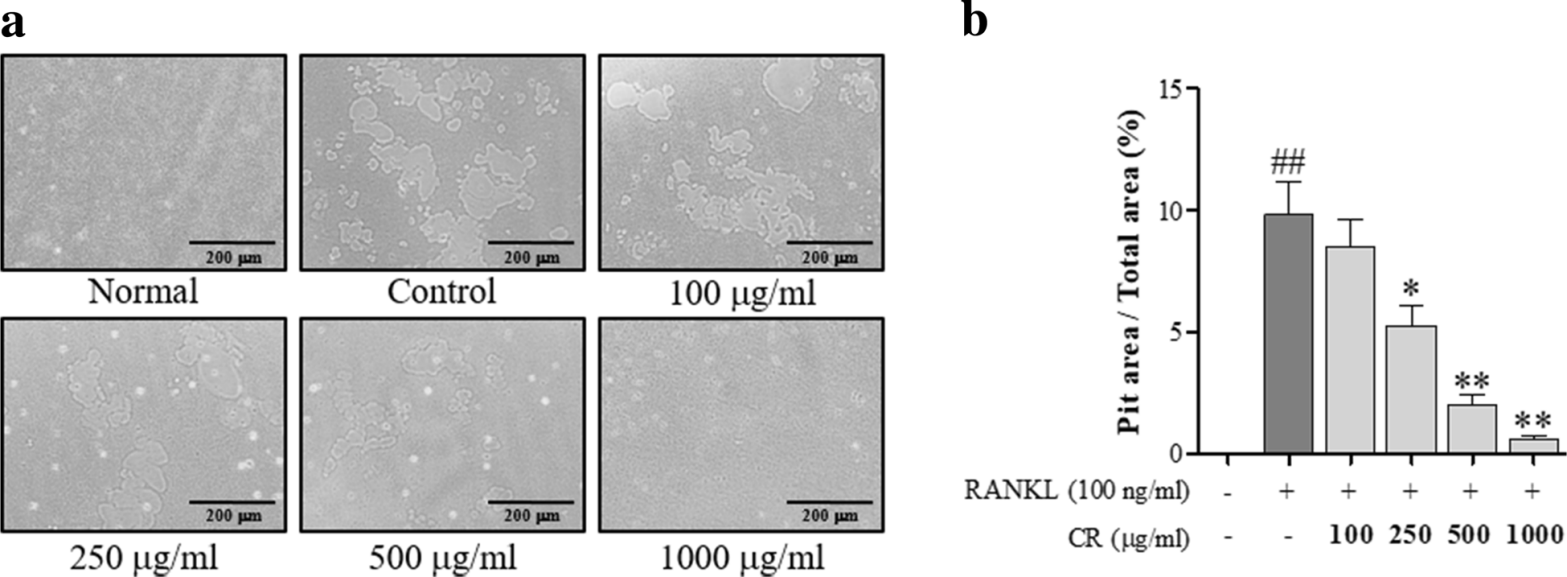
Effect of CR on pit formation. **a** Pit area was observed under an inverted microscope (100×, Scale bars: 200 μm), and **b** the Pit area was measured using the ImageJ software. Columns and error bars represent the mean ± S. E of three independent experiments. ^##^*p* < 0.01 compared with normal and ^**^p < 0.01, ^*^p < 0.05 compared with the control

To determine the effect of CR on the bone resorptive activity, which is a major function of osteoclasts, RAW 264.7 cells were cultured in an osteo strip well plate. CR had an inhibitory effect on bone resorption (Fig. 4a). The measured area was also significantly reduced in the CR treat group (Fig. 4b). These data show that it has an inhibitory effect on the bone resorptive activity.

### CR inhibited NFATc1 and c-Fos protein expression

**Fig. 5:**
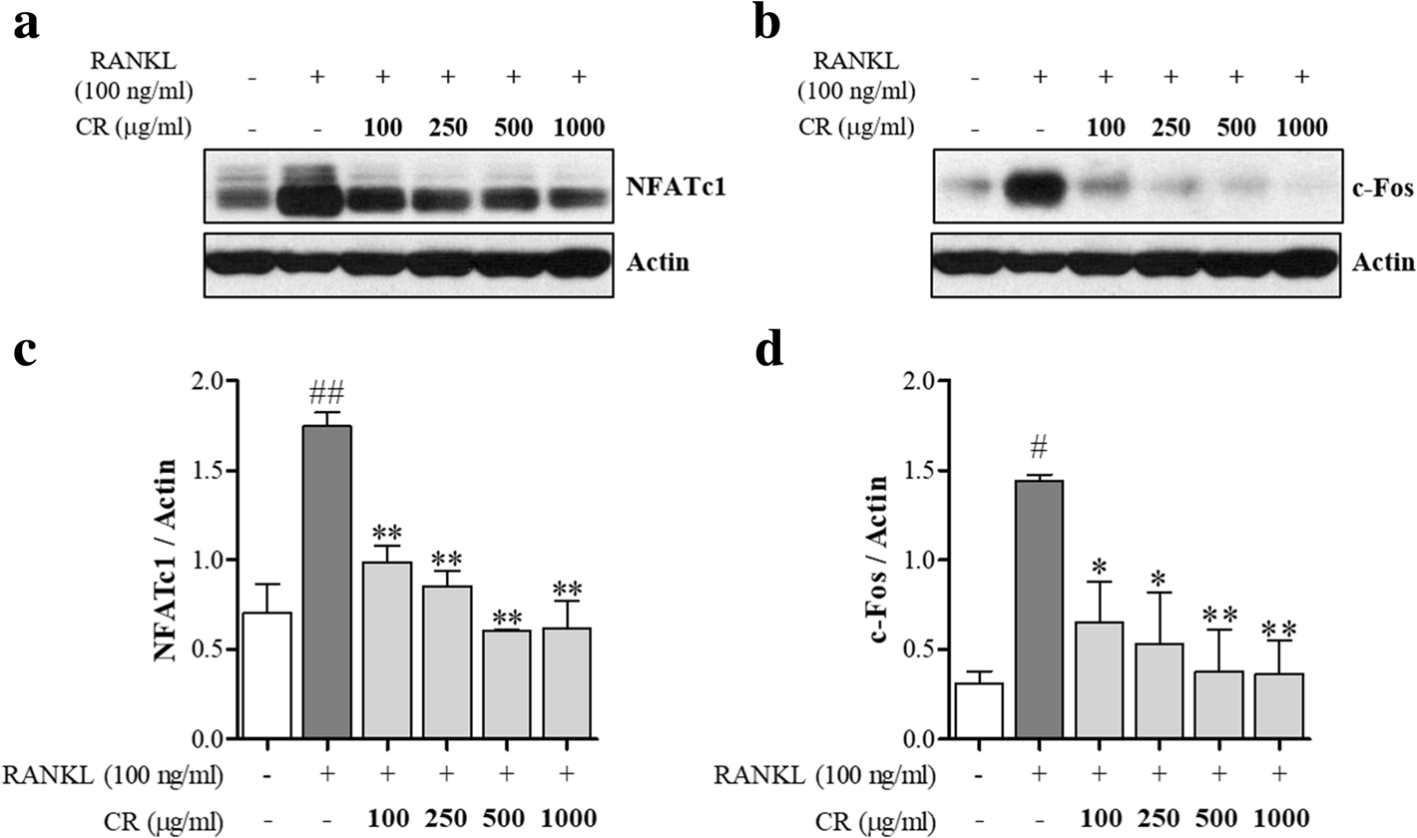
Effect of CR on the activation of NFATc1 and c-Fos by RANKL. **a** NFATc1 and **b** c-Fos protein expressions were determined by western blot. Expressions of **c** NFATc1 and **d** c-Fos were normalized to actin. Columns and error bars represent the mean ± S. E of three independent experiments. ^##^*p* < 0.01, ^#^*p* < 0.05 compared with normal and **p < 0.01, *p < 0.05 compared with the control

The effect of CR on essential osteoclast differentiation indicators, which are NFATc1 and c-Fos, was investigated. NFATc1 is controlled by c-Fos as a master transcription factor for osteoclast differentiation. CR had significant inhibitory effects on NFATc1 protein expression (Fig. 5a); CR also significantly inhibited the c-Fos protein expression (Fig. 5b).

### CR inhibited osteoclastogenesis-related genes

**Fig. 6:**
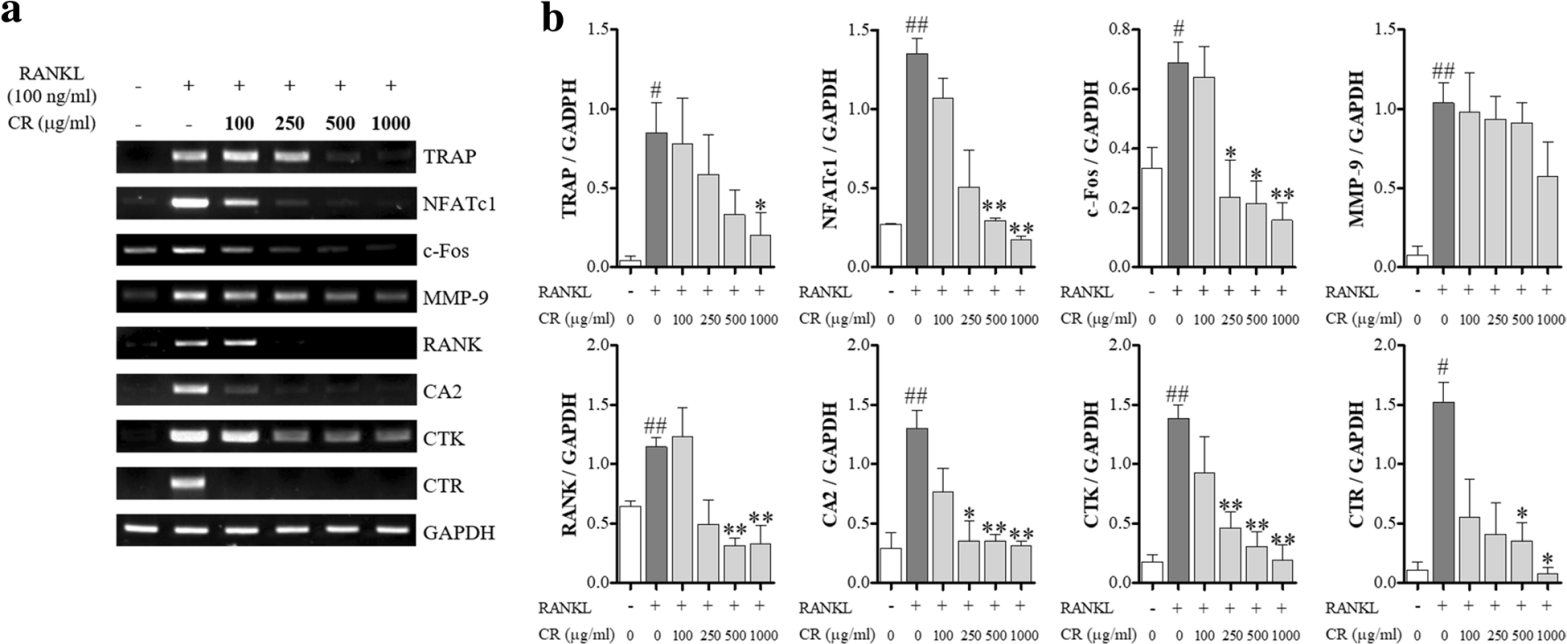
Effect of CR on the mRNA expression of osteoclastogenesis marker genes by RANKL. **a** The mRNA expressions were detected by RT-PCR. **b** osteoclastogenesis genes were normalized to GAPDH. Columns and error bars represent the mean ± S. E of three independent experiments. ^##^*p* < 0.01, ^#^*p* < 0.05 compared with normal and **p < 0.01, *p < 0.05 compared with the control

Next, the effect of CR on the mRNA expression of c-Fos and NFATc1 was explored. We also looked at the effect of CR on the activation of osteoclastogenesis marker by RANKL, such as RANK, TRAP, CTK, CTR, MMP-9, and carbonic anhydrase 2 (CA2). CR inhibited the expression of all osteoclastogenesis-related genes by RANKL in a dose-dependent manner (Fig. 6a). In particular, TRAP, c-Fos, CA2, and CTR were effectively inhibited at low concentrations of CR.

### CR increased the femur weight in the OVX rat model

**Fig. 7:**
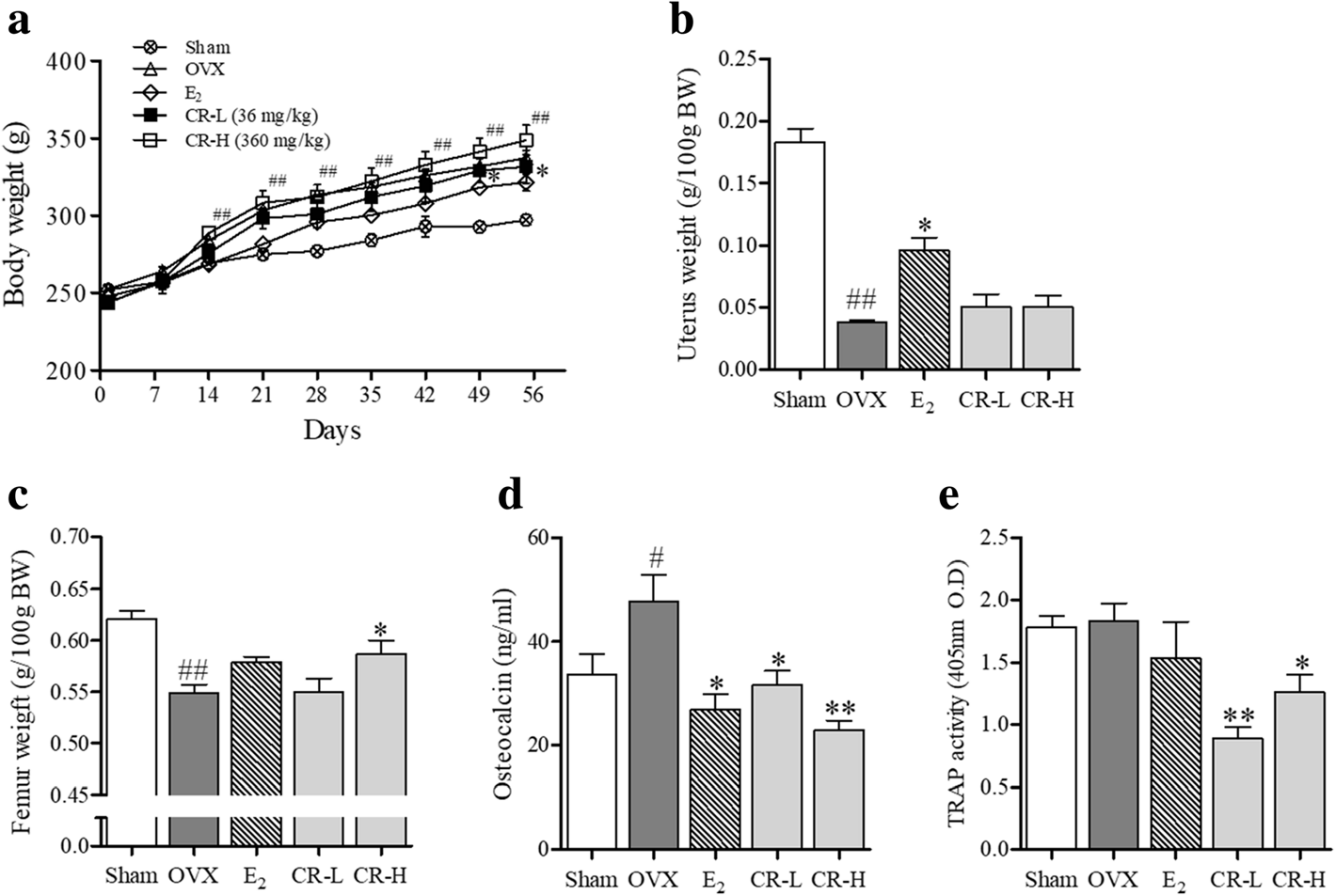
Effect of CR on the OVX-induced osteoporosis rat-model. **a** Weekly body weight of OVX-induced SD-rats, **b** the rats were sacrificed after weighing the uterus and **c** tibia. **d** Osteocalcin and **e** TRAP activity on serum were measured. Columns and error bars represent the mean ± S. E of eight independent experiments. ^##^*p* < 0.01 compared with the Sham and ^*^p < 0.05 compared with OVX

We investigated whether CR suppresses bone loss of OVX rats. After OVX, the OVX group weighed more than the sham group, and the E_2_ group prevented weight gain via OVX. However, CR groups did not affect (Fig. 7a). Uterine weight decreased through OVX, E_2_ group suppressed uterine weight loss, but CR groups showed no changes (Fig. 7b). There was no significant change in the femur weight loss in the E_2._ However, the CR-H group significantly inhibited OVX-induced weight loss of the femur (Fig. 7c).

### CR reduced the serum level of osteocalcin and TRAP activity

To measure the effect of CR treatments on bone turnover markers in OVX rats, we analyzed level of osteocalcin and TRAP in serum. Level of osteocalcin increased by OVX. CR and E_2_ groups had significant inhibitory effects on osteocalcin expression. In particular, the CR-H group had a strong inhibitory effect on osteocalcin (Fig. 7d). As a result of measuring TRAP activity in serum, CR treated group showed suppressive effect (Fig. 7e).

### CR had an inhibitory effect on trabecular loss and osteoclastogenesis in the histologically stained femur

**Fig. 8:**
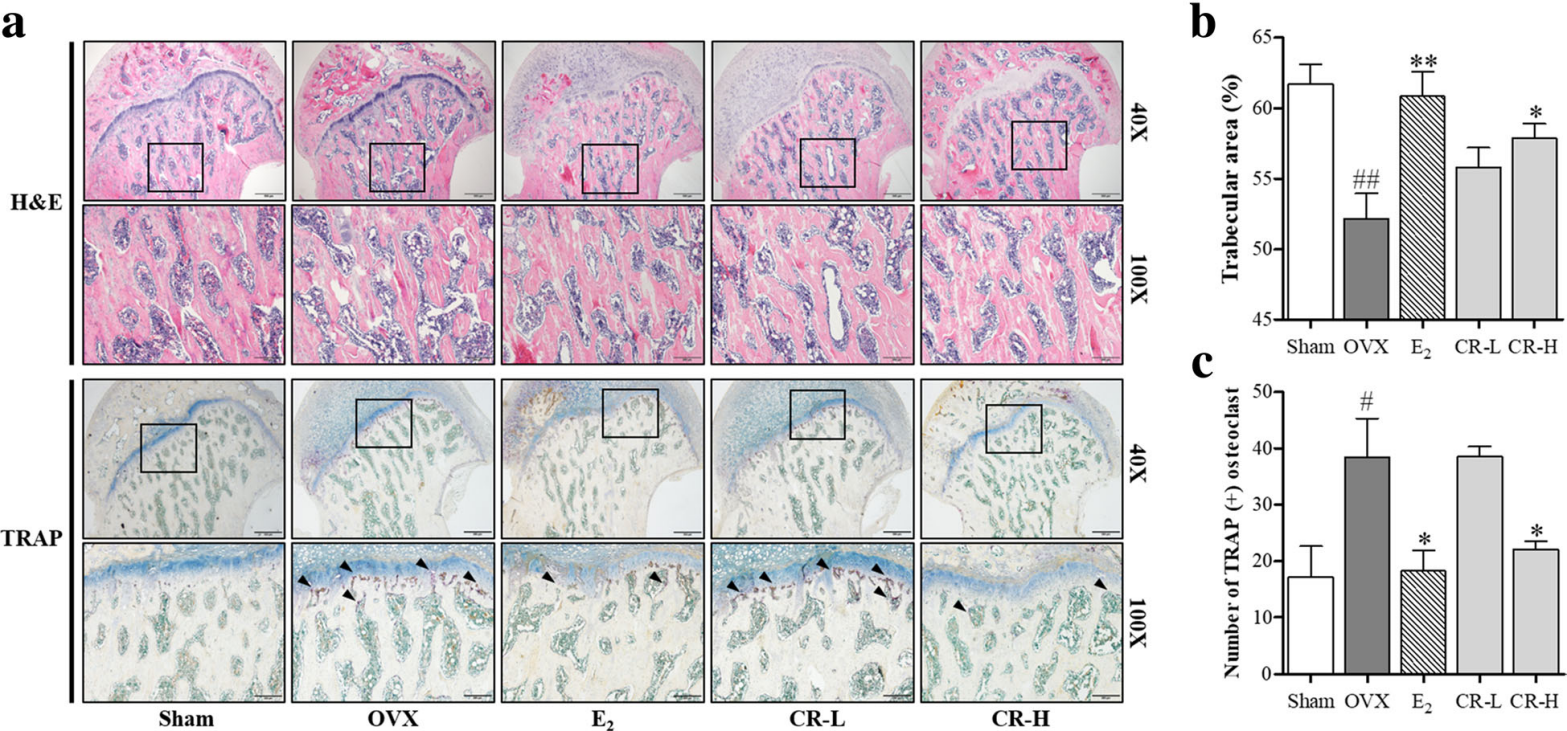
Effect of CR on the trabecular area in OVX-induced bone loss in rats. Histological examination of tissue sections from the femoral heads of rats using H&E and TRAP staining. **a** One representative femur from each group was captured using a light microscope, and the arrowheads indicate the osteoclasts. (40X and 100X magnification, Scale bars are 500 and 200 μm, respectively). **b** Trabecular area was measured using the Image J software and **c** number of osteoclasts was counted using the Image J software. Data represent the mean ± S. E of each experimental groups. ^##^*p* < 0.01, ^#^*p* < 0.05 compared with the Sham and ^**^p < 0.01, *p < 0.05 compared with OVX

To investigate the effect of CR on bone loss and the number of osteoclasts, we did histological staining of the femurs using H&E and TRAP staining. As shown in Fig. 8a, OVX reduced the trabecular area of the femoral bone. Compared to the sham group, the difference is significant and CR-H and E_2_ groups significantly inhibit trabecular area reduction (Fig. 8b). Additionally, the OVX group had a significantly increased number of osteoclasts compared with the sham group. The E_2_ and CR-High groups had a significantly decreased number of osteoclasts compared with the OVX group (Fig. 8c). These data consistently show the inhibitory effects of CR on osteoporosis in the OVX-rat model through the inhibition of osteoclast differentiation.

### CR had an inhibitory effect on the expression of NFATc1, c-fos and CTK expression in femur

**Fig. 9:**
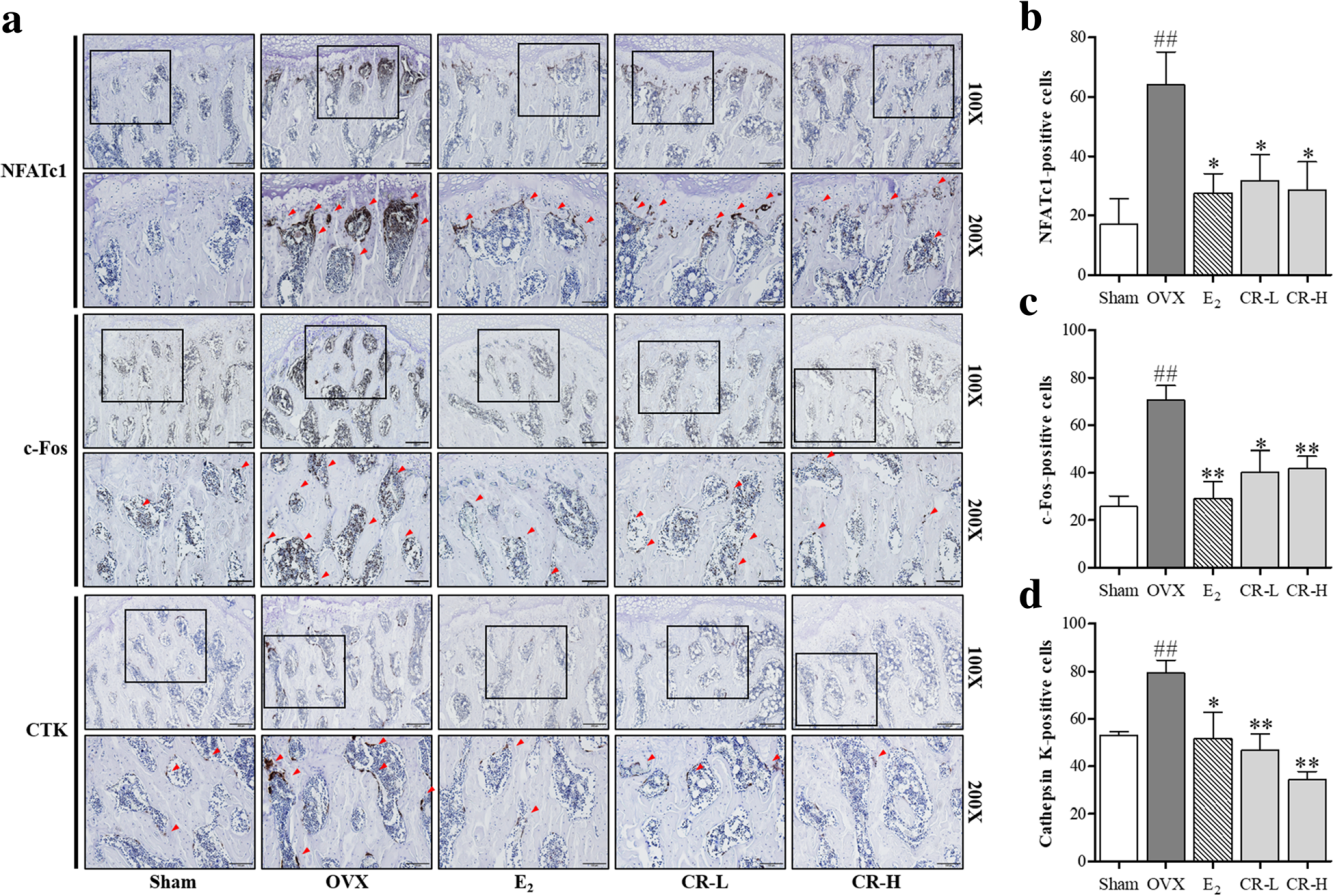
Effect of CR on the expression of NFATc1, c-Fos and CTK in OVX-induced femur. **a** Immunohistochemistry staining of NFATc1, c-Fos and CTK was conducted. One representative femur from each group was captured using a light microscope. The red arrowheads indicate each indicators-positive cells. (100X and 200X magnification, Scale bars are 200 and 100 μm, respectively). **b** Quantification of NFATc1-positive cells, **c** c-Fos-positive cells and **d** CTK-positive cells in femur were examined. Data represent the mean ± S. E of each experimental groups. ^##^*p* < 0.01 compared with the Sham and ^**^p < 0.01, *p < 0.05 compared with OVX

To examine the effect of CR on the expression of NFATc1, c-fos and CTK in the femur, IHC staining was performed. As shown in Fig. 9, compared to the sham group, the expression of NFATc1, c-Fos and CTK increased in the OVX group. CR-L and CR-H groups inhibited expression of NFATc1, c-Fos and CTK in the osteoporotic femur. These results suggest that the anti-osteoporotic effect of CR is due to the suppression of NFATc1, c-Fos and CTK expression in the femur.

## Discussion

In the present study, we demonstrated that CR is a potent inhibitor of osteoclast differentiation in RAW 264.7 cells by the suppression of important transcription marker. Moreover, CR inhibited bone loss and osteoclast differentiation in the OVX rat models. The key causal factor of osteoporosis is abnormal bone resorption of osteoclasts. The inhibition of osteoclast differentiation would be a significant treatment strategy for osteoporosis.

It is important that TRAP staining and activity assays are used when identifying the osteoclast phenotype. To examine the effects of CR on the osteoclast differentiation, RANKL-induced models were used in RAW 264.7 cells [31]. In the present study, CR inhibited osteoclastogenesis and its activity. Osteoclasts are large multinucleated cells with the potential to form resorption lacunae on the bone. Functionally, the pit formation assay is required when identifying the bone resorption activity of osteoclast and the mRNA expression of TRAP, CTK, MMP-9, and CA2 is also important because these genes are involved in the bone resorption [13, 32–34]. To confirm the effects of CR on bone resorption, the bone resorption-related genes were measured by RT-PCR. We found that CR inhibited the pit formation and reduced the RANKL-induced production such as TRAP, CTK, MMP-9, and CA2 genes. These results suggest that CR has an inhibitory effect on bone resorption, which is the main function of osteoclasts.

Previously, many studies have confirmed that NFATc1 is the transcriptional factor involved in T-cell maturation, and it has been reported recently to be the master switch regulator for osteoclast formation and function [5, 15, 35]. Other studies have found that even in the absence of RANKL, overexpression of NFATc1 induces osteoclast precursor cells differentiate into osteoclasts. [15]. In addition, NFATc1 regulate various phenotype genes involved in osteoclastogenetsis and bone resorption such as TRAP, CTK, MMP-9, and CTR [13, 15]. In this study, CR inhibited the mRNA and protein expressions of NFATc1. These data indicate that CR suppresses the mRNA expression of osteoclast-related markers through the inhibition of NFATc1. Recently, it has been reported that c-Fos is a key regulator of osteoclastogenesis and bone remodeling [12]. The removal of the gene encoding c-Fos causes defective osteoclast differentiation and osteopetrosis [36]. Whereas the overexpression of c-Fos in osteoclast progenitors improves osteoclastogenesis [37]. In the present study, CR inhibited the mRNA and protein expressions of c-Fos. These results indicate that CR inhibits both NFATc1 and c-Fos, the key factors of this mechanism, and suppresses the differentiation into osteoclasts. c-Fos also regulates osteoclastogenesis-related genes, such as CA2. The promoter of the gene that encodes CA2 is directly regulated by c-Fos overexpression [34]. CA2 is located on the bone surface and acidifies the surface. After that, bone resorption markers lead to absorb [9, 13, 34, 36]. In this study, CR suppresses CA2 through the inhibition of c-Fos. In addition, we also confirmed the expression of RANK, an early mechanism of RANKL-induced osteoclast [5]. CR also inhibited RANK, these results indicate that the effect of CR to inhibit osteoclast differentiation is a result of down-regulation of RANK on the surface of osteoclast precursor cells (Additional file 1).

Bilateral OVX is a generally used experimental method to recapitulate bone remodeling disorders in animal models [38]. As a result of OVX, the increasing osteoclast activity and osteoclastogenesis is the main mechanism reported to result in bone loss in these models [39]. Lack of estrogen through OVX is caused by a unique atrophy of the uterus [40]. In this study, uterine weight decreased after OVX, it is evidence of the success of OVX and supports the results of other research [41, 42]. CR treatment resulted in no changes in uterus weight compared with the OVX group. Moreover, another characteristic of OVX is an increased body weight; CR had no effect on body weight. The mechanism of OVX and body weight is unclear; however, it is expected to be associated with estrogen deficiency. In contrast, the E_2_ group showed smaller decreases in uterus weight and smaller increases in body weight. These data show that CR does not affect hormone function, such as estrogen [43]. In addition, the induced osteoclasts in the bone increase the concentration of TRAP in the serum. Thus, the serum concentration of osteocalcin, a bone turnover marker, also is increased [44, 45]. In this study, CR significantly inhibited both indicators in serum. TRAP activity was slightly increased by OVX, but the difference was not significant. Although we can’t accurately account for the small difference in TRAP activity between Sham and OVX, it is assumed that the experiment period is short [46]. However, we founded that the CR group had inhibitory effect of TRAP activity. These results indicate that CR inhibits the induction of OVX induced osteoporosis.

Our study also showed that CR reduced bone loss in an OVX rat model [47]. The bone histological examination results indicated that OVX increased the bone resorption area and decreased the bone weight [9]. Low bone mass is a major risk factor for fractures, and OVX significantly increased the bone trabecular area [2, 38]. In this study, the CR-High group showed an inhibitory effect with a reduced trabecular area. Additional, CR inhibited osteoclast differentiation and expression of NFATc1, c-Fos and CTK in femoral tissue. These data indicate that CR is a beneficial treatment for postmenopausal osteoporosis by through inhibition of osteoclast differentiation through inhibition of NFATc1.

## Conclusion

In conclusion, our findings clearly show that CR has an inhibitory effect on osteoclastogenesis by RANKL. CR inhibited the expression of osteoclastogenesis markers, such as TRAP, CTK, CTR, MMP-9, RANK, CA2, NFATc1 and c-Fos. Moreover, CR also decreased the OVX-induced bone loss and expression of NFATc1 and CTK in femur. Thus, our results indicate that CR may have potential of a therapeutic herb for bone diseases associated with abnormal osteoclast formation and bone destruction. However, further research is needed, with a particular focus on potential side effects.

## Additional file

Additional file 1: Scheme for CR inhibition of RANKL-induced oateoclastogenesis (├, ↓: inhibition). (TIF 670 kb)

## Data Availability

All data generated or analysed during this study are included in this published article [and its supplementary information files].

## Abbreviations

CA2: Carbonic anhydrase 2
CR: Cnidii Rhizoma
CTK: Cathepsin K
CTR: Calcitonin receptor
DMEM: Dulbecco’s medium Modified Eagle
ECL: Enhanced chemiluminescence
GAPDH: Glyceraldehyde 3-phosphate dehydrogenase
H&E: Hematoxylin–eosin
HPLC: High performance liquid chromatography
IHC: Immunohistochemistry
MMP-9: Matrix metallopeptidase-9
NFATc1: Nuclear factor of activated T-cells, cytoplasmic 1
NF-κB: Nuclear factor kappa B
OVX: Ovariectomized
RANK: Receptor activator of the nuclear factor kappa B
RANKL: Receptor activator of the nuclear factor kappa B ligand
RT-PCR: Reverse transcription polymerase chain reaction
TRAF6: Tumor necrosis factor receptor-associated factor 6
TRAP: Tartrate-resistant acid phosphatase
