# Supplementary figures and images for: Water extract of Cnidii Rhizoma suppresses RANKL-induced osteoclastogenesis in RAW 264.7 cell by inhibiting NFATc1/c-Fos signaling and prevents ovariectomized bone loss in SD-rat
Source: BMC Complement Altern Med. 2019 Aug 9;19:207. doi: 10.1186/s12906-019-2611-8 (PMC6688344; doi:10.1186/s12906-019-2611-8)

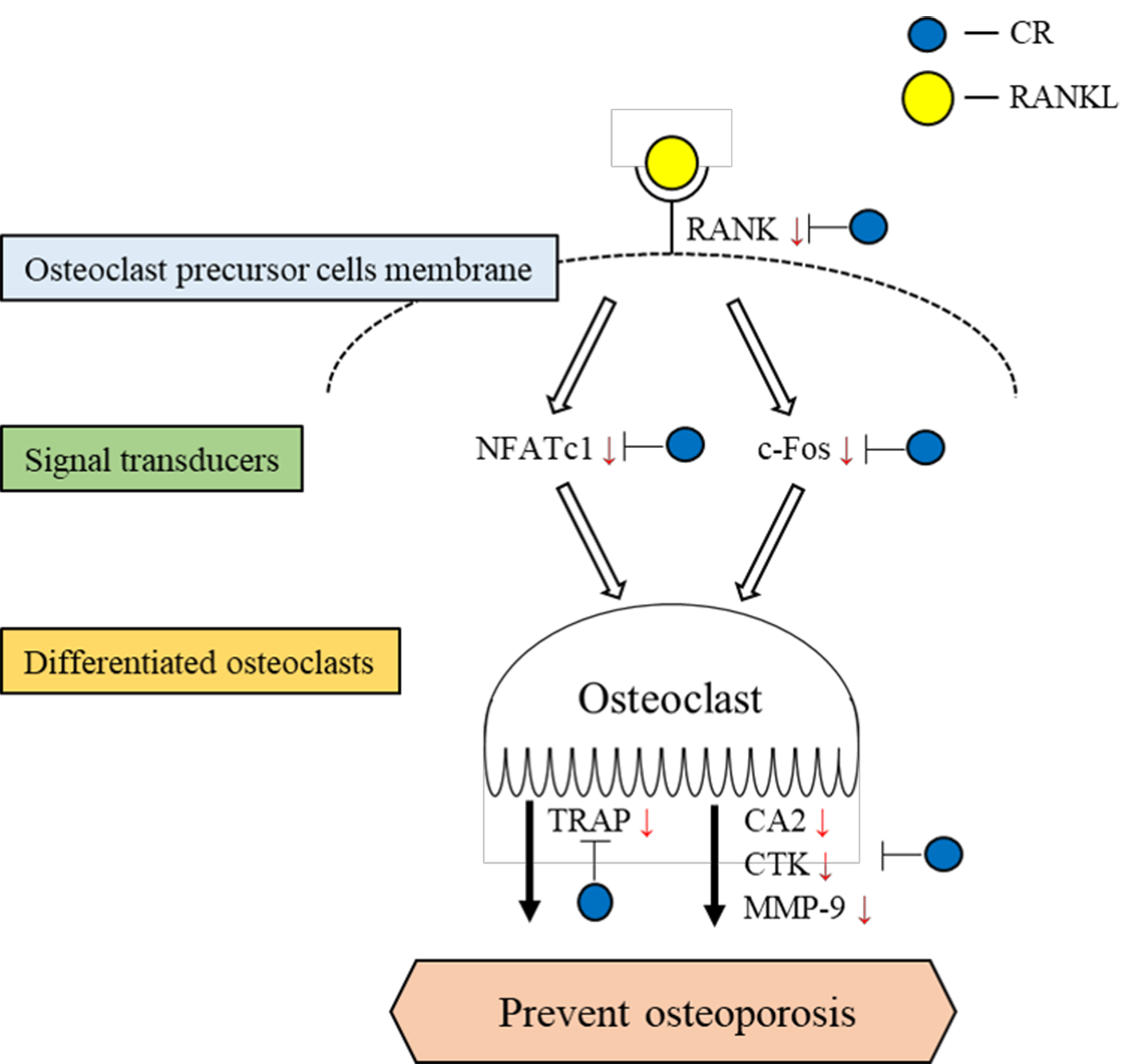

Supplement: Supplementary file 1 — Scheme for CR inhibition of RANKL-induced oateoclastogenesis (├, ↓: inhibition). (TIF 670 kb) [file 12906_2019_2611_MOESM1_ESM.tif]
